# Dipeptidyl‐peptidases: Key enzymes producing entry forms of extracellular proteins in asaccharolytic periodontopathic bacterium *Porphyromonas gingivalis*

**DOI:** 10.1111/omi.12317

**Published:** 2020-10-12

**Authors:** Takayuki K. Nemoto, Yuko Ohara Nemoto

**Affiliations:** ^1^ Department of Oral Molecular Biology Course of Medical and Dental Sciences Nagasaki University Graduate School of Biomedical Sciences Nagasaki Japan

**Keywords:** diabetes mellitus, exopeptidase, oligopeptide transporter, periodontal disease, acylpeptidyl‐oligopeptidase, oligopeptide transporter

## Abstract

*Porphyromonas gingivalis*, a pathogen of chronic periodontitis, is an asaccharolytic microorganism that solely utilizes nutritional amino acids as its energy source and cellular constituents. The bacterium is considered to incorporate proteinaceous nutrients mainly as dipeptides, thus exopeptidases that produce dipeptides from polypeptides are critical for survival and proliferation. We present here an overview of dipeptide production by *P. gingivalis* mediated by dipeptidyl‐peptidases (DPPs), e.g., DPP4, DPP5, DPP7, and DPP11, serine exopeptidases localized in periplasm, which release dipeptides from the N‐terminus of polypeptides. Additionally, two other exopeptidases, acylpeptidyl‐oligopeptidase (AOP) and prolyl tripeptidyl‐peptidase A (PTP‐A), which liberate N‐terminal acylated di‐/tri‐peptides and tripeptides with Pro at the third position, respectively, provide polypeptides in an acceptable form for DPPs. Hence, a large fraction of dipeptides is produced from nutritional polypeptides by DPPs with differential specificities in combination with AOP and PTP‐A. The resultant dipeptides are then incorporated across the inner membrane mainly *via* a proton‐dependent oligopeptide transporter (POT), a member of the major facilitator superfamily. Recent studies also indicate that DPP4 and DPP7 directly link between periodontal and systemic diseases, such as type 2 diabetes mellitus and coagulation abnormality, respectively. Therefore, these dipeptide‐producing and incorporation molecules are considered to be potent targets for prevention and treatment of periodontal and related systemic diseases.

**Abbreviations**
AOP: acylpeptidyl‐oligopeptidase
Dcp: peptidyl‐dipeptidase
DPP: dipeptidyl‐peptidase
GIP: glucose‐dependent insulinotropic polypeptide
GLP‐1: glucagon‐like peptide 1
MCA: 4‐methycoumaryl‐7‐amide
OPT: oligopeptide transporter
POT: proton‐dependent oligopeptide transporter
PTP‐A: prolyl tripeptidyl‐peptidase A
SstT: serine/threonine transporter

## 1 INTRODUCTION

Periodontal disease, a bacterial‐associated inflammatory condition that occurs around the gingivae, is a leading cause of tooth loss in adults, with 20%–50% of individuals affected worldwide, which results in a decrement in overall quality of life especially in elderly individuals (Nazir, 2017). A large number of epidemiological studies have shown a keen association of chronic periodontitis and type 2 diabetes mellitus (Grossi & Genco, 1998; Lalla & Papapanou, 2011; Preshaw et al., 2012), and recently much attention has been given to this oral disease because of its close relationship to systemic diseases, such as atherosclerotic cardiovascular disorder (Genco & VanDyke, 2010; Tabeta et al., 2014), decreased kidney function (Kshirsagar et al., 2007), rheumatoid arthritis (Detert et al., 2010), and Alzheimer's disease (Dominy et al., 2019; Teixeira et al., 2017).

Periodontal disease is initiated by a complex of bacterial species that form subgingival biofilm in and around the gingival crevice. When allowed to progress, inflammatory cytokines and chemokines are released from affected tissues, implying a connection of this oral inflammatory condition to other systemic diseases. In previous studies, Socransky and Haffajee and their colleagues stratified the microbiota into five major complexes, among which the first one, designated “red complex” and comprised of *Porphyromonas gingivalis*, *Tannerella forsythia,* and *Treponema denticola*, is strikingly associated with clinical measures of periodontal disease, and thus those members are considered to be major periodontopathic bacteria implicated in severe forms (Holt & Ebersole, 2005; Socransky & Haffajee, 2002; Socransky et al., 1998). Whole genome analysis has suggested that *P. gingivalis* can metabolize several sugars including melibiose and galactose (Nelson et al., 2003). Nevertheless, *P. gingivalis* and *T. forsythia* are in practical terms asaccharolytic and their growth is supported by proteinaceous substrates. Sugar utilization may be not so beneficial for periodontal bacteria, because sugar supply is quite limited in their niche. This feature of utilization of amino acids as the sole carbon and energy source is quite singular among oral microorganisms and seems to be advantageous for settlement of these pathogenic bacteria in subgingival plaque. However, until recently little has been reported regarding how nutritional amino acids are produced from extracellular proteins and become incorporated into cells of those bacteria.

Extracellular proteins in *P. gingivalis* are initially degraded into oligopeptides by the well‐characterized cysteine endopeptidases Arg‐gingipains (Kadowaki et al., 1994; Pavloff et al., 1995) and Lys‐gingipain (Pavloff et al., 1997; Scott et al., 1993), which are localized in the outer membrane and outer membrane vesicles. The resulting oligopeptides are transported into the periplasmic space. As they are still larger than the size incorporated across the inner membrane through the outer membrane, they must be further trimmed by exopeptidases prior to incorporation.

Collateral evidence has indicated that *P. gingivalis* incorporates dipeptides derived from proteinaceous nutrients. Tang‐Larsen et al. (1995) reported that additions of Met‐Met and Cys‐Cys enhanced the production of methyl mercaptan and hydrogen sulfide, respectively, whereas neither a single Met nor Cys showed increased production. The dominance of dipeptides as nutrients in *P. gingivalis* was also demonstrated by findings showing significant accumulation of their metabolites, such as ammonia and short‐chain fatty acids (Takahashi & Sato, 2001, 2002). These findings strongly suggest that not a single amino acid but dipeptide is the major form incorporated into *P. gingivalis* cells. In accordance with this notion, a series of dipeptide‐producing exopeptidases, dipeptidyl‐peptidases (DPPs), have been discovered in *P. gingivalis*.

In this article, we discuss the roles of DPPs and two additional exopeptidases in dipeptide production, as well as the specific distribution of *dpp* genes in periodontopathic bacteria. Furthermore, we demonstrate the importance of a proton‐dependent oligopeptide transporter (POT) in dipeptide incorporation in *P. gingivalis*. The relationship of bacterial DPPs with systemic diseases is also discussed.

## 2 DEGRADATION OF EXTRACELLULAR PROTEINS INITIATED BY GINGIPAINS

Gingipains are cysteine proteinases that consist of Lys‐specific (Lys‐gingipain/Kgp) (Pavloff et al., 1997; Scott et al., 1993) and two Arg‐specific (Arg‐gingipains/Rgps) proteinases (Pavloff et al., 1995). Gingipains degrade various human proteins, including complement system proteins, cytokines, and integrin (O'Brien‐Simpson et al., 2001), and their roles as virulence factors have been reviewed elsewhere (Fitzpatrick et al., 2009; Kadowaki et al., 2007; Potempa et al., 2003). The triple‐gingipain gene null mutant KDP136 was shown unable to grow in a defined medium with human albumin as the sole carbon source (Shi et al., 1999), implicating that gingipains are not only virulence factors but also essential for bacterial growth.

Following degradation of extracellular proteins by gingipains, the resultant oligopeptides are imported into periplasmic space by likely an import mechanism. Madej et al., 2020) recently reported the molecular structure of RagAB, the cell wall importer for oligopeptides, and showed that the RagAB dimer forms a large internal cavity, which seems adaptive for accepting oligopeptides from 7 to 29 residues in length.

### 2.1 DIPEPTIDE PRODUCTION MEDIATED BY DPPs

DPPs are exopeptidases that liberate dipeptides from the unblocked N‐terminus of oligopeptides and proteins, with eight members classified by amino acid sequence similarity and substrate specificities; DPP1–6 (or I–VI), 7, and 11. Roman numerals have been used for DPPI‐DPPVI, though for simplicity and to follow more recent usage, this study uses Arabic numbers for all DPPs. Other DPP members with various designations, *i.e*., QPP, FAPα (Retting et al., 1994), DPP6, DPP8, and DPP9 (Olsen & Wagtmann, 2002), DPP10 (Qi et al., 2003), and DPP‐A and DPP‐B are subtypes of eukaryotic DPP4, because of their sequence similarities (Table 1).

**TABLE 1:** DPPs and two related exopeptidases expressed in *P. gingivalis*

Name	IUBMB	MEROPS code	Gene code^a^	Specificity^b^	Synthetic and native substrate	Isozyme	Reference
DPP3	EC3.4.14.4	M49.001	PGN_01645	NH_2_‐RR‐|‐	RR‐NHMec		Ellis & Nuenke (1967); Ohara‐Nemoto et al. (2014)
DPP4	EC3.4.14.5	S9.013	PGN_1469	NH_2_‐XP‐|‐ NH_2_‐XA‐|‐	GP‐MCA Incretin	FAPα/seprase DPP6/DPPX DPP8/DPRP1 DPP9/DPRP2 DPP10/DPRP3 DPP‐A and B	Banbula et al. (2000); Ohara‐Nemoto et al. (2017)
DPP5		S9.012	PGN_0756	NH_2_‐AA‐|‐ NH_2_‐HS‐|‐ NH_2_‐SY‐|‐	KA‐MCA		Ohara‐Nemoto et al. (2014)
DPP6		C40.001	PGN_0754	L‐Ala‐γ‐D‐Glu‐|‐			Guinand et al. (1979)
DPP7		S46.001	PGN_1479	NH_2_‐AF‐|‐ NH_2_‐ZZ‐|‐	FM‐MCA		Banbula et al. (2001); Rouf, et al. (2013); Nemoto et al. (2018)
DPP11		S46.002	PGN_0607	NH_2_‐ZD‐|‐ NH_2_‐ZE‐|‐	LD‐MCA LE‐MCA		Ohara‐Nemoto et al. (2011); Rouf, et al. (2013); Nemoto et al. (2017)
AOP		S9C	PGN_1349	Acyl‐ZZ‐|‐ Acyl‐XZZ‐|‐	Benzyloxycarbonyl‐KM‐MCA		Nemoto et al. (2016); Nemoto et al. (2019)
PTP‐A	EC3.4.14.12	S9.017	PGN_1149	NH_2_‐XXP‐|‐	GAP‐β‐naphthylamide		Banbula et al. (1999); Ito et al. (2006)

^a^ *P. gingivalis* ATCC 33277 (Naito et al., 2008).

^b^ X, any amino acid, Z, hydrophobic amino acid, “‐|‐”, cleavage site

The substrate specificity of DPPs is primarily defined based on the penultimate amino acid residue from the N‐terminus (P1 position), and the N‐terminal residue (P2‐position) has effects to some extent on activity towards *P. gingivalis* DPP7 and DPP11 (Rouf, et al., 2013). Pro at the P1’‐position, the C‐terminal side residue of the hydrolyzed peptide bond, is never accepted by any DPP, thus peptides with NH_2_‐Xaa1‐Xaa2‐Pro3‐ are resistant to all DPPs. P1’‐position and its C‐terminal adjacent P2’‐position residues, except for Pro, are least involved in substrate recognition. Structural analysis of human DPP4 revealed a limited interaction with P1’‐position Ser and P2’‐position Lys of YP/SKPDNPGE, first ten residues of neuropeptide (Aertgeerts et al., 2004). Similarly, P1’‐ and P2’‐position residues of Leu‐Asp/‐Val‐Trp exhibit few interactions with *P. endodontalis* DPP11 (Bezerra et al., 2017). These findings indicate that both P1’ and P2’ residues are not tightly associated with DPP4 and DPP11, and presumably other DPPs as well. Polypeptides with N‐terminal modification are not acceptable for DPPs. In *P. gingivalis* and human DPP4, this is due to the necessity of the ionic interaction between the N‐terminal α‐amino group of the substrate and carboxy groups of Glu195 and Glu196 (Rea et al., 2017).

*Porphyromonas gingivalis* possesses six DPPs, *i.e*., DPP3–7, and DPP11. A cross‐search of the oral bacterial and homology databases indicated that 43 species of 772 taxa possess DPP4, DPP5, DPP7, and DPP11 genes (Ohara‐Nemoto et al., 2018). Major bacterial species belong to the genera *Bacteroides*, *Porphyromonas*, *Prevotella, Tannerella*, and *Capnocytophaga* in the phylum *Bacteroidetes*, indicating that all or most of these DPPs are specifically distributed in anaerobic and facultative anaerobic oral rods, which are likely included in subgingival biofilm (Ohara‐Nemoto et al., 2018). In fact, DPP4, DPP5, DPP7, and DPP11 activities have been detected in *Porphyromonas endodontalis* (Nishimata et al., 2014), *Tan. forsythia*, and *Pre. intermedia*, as well as *P. gingivalis* and subgingival plaque specimens (Ohara‐Nemoto et al., 2018).

### 2.2 DPP3

DPP3 belongs to the M49.001 peptidase family and is widely distributed among eukaryotic (Ellis & Nuenke, 1967) and prokaryotic organisms, and is known to readily hydrolyze NH_2_‐Arg1‐Arg2‐|‐Xaa3‐. *P. gingivalis* DPP3 exhibits a 19.0% amino acid identity to the human entity and shows the highest activity for a synthetic substrate, Arg‐Arg‐4‐methycoumaryl‐7‐amide (MCA), followed by Leu‐Arg‐, Ala‐Arg‐, and Lys‐Ala‐MCA, the same as the eukaryotic entity (Ohara‐Nemoto et al., 2014). *P. gingivalis* DPP3 is composed of 906 and 886 amino acid residues in the strains ATCC 33277 and W83, respectively, which are substantially larger than 675 residues of *Bacteroides thetiotaomicron* DPP3 and 737 residues of human DPP3 due to the C‐terminally tagged sequence. The additional sequence is predicted at high confidence to have α‐α superhelix fold, belonging to Armadillo‐type fold family similar to the AlkD family of bacterial glycosylases (Hromić‐Jahjefendić et al., 2017). The genes encoding DPP3 with AlkD domain are specifically found within the genus *Porphyromonas*.

There are two lines of evidence indicating that *P. gingivalis* DPP3 is not responsible for dipeptide production from nutritional proteins. First, it does not contain a signal sequence, thus seems to be localized in cytoplasm, and second, *P. gingivalis* cells show little hydrolysis for Arg‐Arg‐MCA (Ohara‐Nemoto et al., 2014). Accordingly, we speculate that DPP3 functions for degradation of cytosolic proteins and that degradation of nutritional peptides at Arg‐Xaa bonds is already completed by Arg‐gingipains.

### 2.3 DPP4

DPP4 is widely distributed in a variety of species from eukaryotic (S9.003) (Misumi et al., 1992) to bacterial (S9.009) (Ahrén et al., 2000). Human DPP4/CD26 hydrolyzes the peptide bond at the carboxyl side of Pro2 most preferentially and Ala2 at a reduced rate. DPP4 is a key factor that modulates postprandial hyperglycemia by degrading incretin peptides, *i.e*., glucagon‐like peptide 1 (GLP‐1) and glucose‐dependent insulinotropic polypeptide (GIP), which induce secretion of insulin from pancreatic β cells. DPP4 inhibitors, a class of oral antidiabetic agents, such as sitagliptin, vildagliptin, and alogliptin, suppress degradation of incretins and improve glucose tolerance and insulin secretion (Ahrén et al., 2000; Gallwitz, 2019; Weber, 2004). Human DPP4 is a type II membrane protein, in which the N‐terminal hydrophobic sequence represents an uncleavable signal peptide, and functions as a membrane‐anchoring domain (Misumi et al., 1992), and the entity expressed in lymphocytes, and endothelial and epithelial cells as a functional receptor for human Middle East respiratory syndrome coronavirus (MERS‐CoV) (Raj et al., 2013).

*Porphyromonas gingivalis* DPP4 exhibits the substrate specificity similar to that of human DPP4 (Banbula et al., 2000) and a very overall structure to human entity (Rea et al., 2017), despite a limited amino acid identity (23.8%) to human DPP4 (Kiyama et al., 1998). Periodopathic bacterial DPP4 as well as human DPP4 degrades incretin and modulates blood glucose levels in mice (Ohara‐Nemoto et al., 2017). This issue will be described in detail in Section 9. *P. gingivalis* DPP4 also degrades substance P, fibrin inhibitory peptide, IL‐1β, IL‐2, and β‐casomorphin in vitro (Banbula et al., 2000). Mice injected with the *P. gingivalis* W83 strain were found to develop abscesses to a greater extent and died more frequently than those challenged with a *dpp4*‐deficient strain (Kumagai et al., 2000). However, this phenomenon may not represent virulence potential, but the nutritional contribution of DPP4 (Ohara‐Nemoto et al., 2014).

Human DPP4 inhibitors, i.e., P32/98, viladagliptin, and sitagliptin, also suppress the activity of periodontopathic bacterial DPP4 at 250 µM (Ohara‐Nemoto et al., 2014). A recent quantitative analysis demonstrated IC_50_ of vildagliptin (1.3 µM) and sitagliptin (18 µM) to human DPP4, which are 1/10.8‐ and 1/450‐fold lower IC_50_ concentrations of vildagliptin and sitagliptin, respectively, required for *P. gingivalis* DPP4 (Rea et al., 2017). These findings suggest that *P. gingivalis* DPP4 entering into the bloodstream is inefficiently inhibited by prescribed DPP4 inhibitors for type 2 diabetes mellitus patients (Rea et al., 2017).

### 2.4 DPP5

DPP5 was initially found in *Aspergillus fumigatus* (Beauvais et al., 1997), then later in *P. gingivalis* as the first bacterial species (Ohara‐Nemoto et al., 2014). Based on the discovery of *P. gingivalis* DPP5, its wide distribution among bacteria and archaea, as well as in eukaryotes including higher animals and plants has been revealed.

The amino acid sequence of *P. gingivalis* DPP5 is 28.5% identical to that of *A. fumigatus* DPP5. *P. gingivalis* DPP5 preferentially removes dipeptides with Ala and hydrophobic residues at the P1 position from synthetic dipeptidyl MCA substrates. Lys‐Ala‐MCA has been identified as the most potent substrate, though no native substrate has yet been identified.

### 2.5 DPP6

DPP6 removes the N‐terminal dipeptide L‐Ala‐D‐Glu by hydrolysis of the γ‐D‐glutamyl‐(L)‐diamino acid bond (Guinand et al., 1979). The substrates L‐Ala‐γ‐D‐Glu‐L‐Zaa‐Y (Zaa = a diamino acid that may be L‐lysine, meso‐diaminopimelic acid, ω‐amidated‐ or meso‐diaminopimelic acid, and Y is either D‐Ala or D‐Ala‐D‐Ala) are the peptide moieties of bacterial peptidoglycans. DPP6 belongs to C40 family, of which the representative is that of *Bacillus sphaericus*. *B. sphaericus* DPP6 is composed of 271 amino acids with no signal peptide, as expected for a cytoplasmic enzyme. The gene of DPP6 (PGN_0754) encoding a 201 amino acid protein is also present in *P. gingivalis*, although there is no biochemical characterization on this gene product. A biochemical analysis is needed to elucidate the role of *P. gingivalis* DPP6.

### 2.6 DPP7

DPP7, a member of S46.001, was initially discovered in *P. gingivalis* and does not exist in eukaryotic organisms. In this respect, it should be noted that human DPP7 is not related to bacterial DPP7, but rather is an isozyme of DPP2 (Bezerra et al., 2012). DPP7 releases the N‐terminal dipeptide NH_2_‐Xaa1‐Zaa2 (Zaa, hydrophobic residues) (Banbula et al., 2001; Rouf, et al., 2013), and hydrolyzes insulin B chain, type I collagen, and azocasein in vitro (Banbula et al., 2001) Since the P1‐position specificity of DPP7 overlaps with that of DPP5, findings of a specific synthetic substrate for DPP7 were eagerly anticipated and Phe‐Met‐MCA finally identified (Nemoto et al., 2018). As a result, it is now possible to distinctly measure all DPP activities expressed in bacteria and clinical specimens with Arg‐Arg‐, Gly‐Pro‐, Lys‐Ala‐, Phe‐Met‐, and Leu‐Asp‐MCA for DPP3, DPP4, DPP5, DPP7, and DPP11, respectively, as well as for determining activities in saliva and subgingival dental plaque specimens. Their activities were successfully determined in an exception of the DPP4 activity of saliva due to the presence of human DPP4 (Ohara‐Nemoto et al., 2018).

Although both DPP5 and DPP7 show a common hydrophobic P1 preference, DPP5 has no apparent amino acid preference at the P2 position (Ohara‐Nemoto et al., 2014), in contrast to the hydrophobic P2 preference of DPP7 (Rouf, et al., 2013). Thus, the difference in P2‐position preference between DPP5 and DPP7 amplifies the repertoire of peptide substrates of *P. gingivalis*. For example, *P. gingivalis* DPP7 hydrolyzes Leu‐Arg‐, Leu‐Gln‐, and Leu‐Glu‐MCA to some extent, while those are scarcely hydrolyzed by DPP5 (Ohara‐Nemoto et al., 2014). Hence, hydrolyzation of Leu‐Gly‐β‐naphthylamine, which has been observed in five *P. gingivalis* strains, is likely mediated by DPP7 (Suido et al., 1986).

### 2.7 DPP11

A metabolite analysis of *P. gingivalis* indicated that glutamic acid/glutamine (Glx)‐ and aspartic acid/asparagine (Asx)‐containing peptides were the most intensively consumed (Takahashi et al., 2000), and those experimental results were supported by computational analysis of the genome‐scale metabolic network using flux balance calculation (Mazumdar et al., 2009). However, how Asp‐ and Glu‐containing dipeptides are produced in the bacterium was not elucidated, until being revealed by the finding of novel Asp‐ and Glu‐specific DPP 11 (Ohara‐Nemoto et al., 2011).

A previous study noted that *P. gingivalis* DPP11 encoded by PGN_0607 in strain ATCC 33277 may be an isozyme of DPP7, because of its 38.4% amino acid identity to DPP7 encoded by PGN_1479 (Banbula et al., 2001). However, it was later shown that the product of PGN_0607 is a novel DPP specific for acidic amino acid residues Asp and Glu, exclusively distinct from the hydrophobic specificity of DPP7, thus DPP11 was classified as an S46.002 peptidase. S46 family peptidases are solely distributed in bacteria, and DPP11 as well as DPP7 prefer hydrophobic residues at the P2 position (Rouf, et al., 2013).

The acidic P1‐position residue is recognized by Arg673 in *P. gingivalis* DPP11. Biochemical studies have revealed that there are three subtypes of DPP11; (a) *Porphyromonas‐*type DPP11, which is Asp‐preferential, (b) *Bacteroides‐*type DPP11, which shows a high preference for Asp and scarcely accepts Glu, and (c) *Shewanella‐*type DPP11, in which S1 Ser673 instead of Arg673, primarily exhibits higher preference for Glu (Nemoto et al., 2017).

In DPP7, Arg673 of DPP11 is replaced by Gly666, which allows the recognition of bulky hydrophobic P1 residue (Ohara‐Nemoto et al., 2011). Furthermore, Gly666Arg substitution in recombinant DPP7 resulted in a partial acquisition of hydrolyzing activity against acidic residues (Rouf, et al., 2013). Also, elucidation of the three‐dimensional structures of a DPP7‐family member, DAP BII, from *Pseudoxanthomonas mexicana* (Sakamoto et al., 2014) and *P. gingivalis* DPP11 (Bezerra et al., 2017; Sakamoto et al., 2015) confirmed the essential positionings of Gly666 of DPP7 and Arg673 of DPP11 for P1‐position residue recognition. Coding sequence annotation tools that adopt sequence similarities can present misleading results regarding the classification of DPP7 and DPP11. Instead, we proposed that two S46‐family members can be readily classified by use of characteristic residues, *i.e*., Gly666 of DPP7 and an equivalent residue Arg673 of DPP11 (Ser673 in *Shewanella*‐type DPP11) (Rouf, et al., 2013) This classification was then successfully used to identify *Capnocytophaga canimorsus* DPP7 as it contains Gly666 (Hack et al., 2017), despite the annotation regarding a DPP11‐like protein in the MEROPS database (Rawlings et al., 2014). Besides, Bezerra et al. (2017) demonstrated a distinct thermodynamic signature in *P. gingivalis* DPP11 showing that protein conformational entropy is the main driving‐force for substrate binding. Since S46‐family members DPP7 and DPP11 are not present in eukaryotic organisms, they are attractive targets for drugs of periodontal disease. In this line, Sakamoto et al. (2019) developed nonpeptidyl DPP11 inhibitor SH‐5 with IC_50_ of 90.1 µM, which showed a dose‐dependent inhibition of the growth of *P. gingivalis*.

## 3 EXOPEPTIDASES FACILITATING DIPEPTIDE PRODUCTION

The existence of multiple DPPs should be beneficial for complete degradation of polypeptides into dipeptides. However, there appear to be two types of peptide bonds resistant to bacterial DPPs identified to date. First, N‐terminally‐acylated polypeptides are resistant to all DPPs as well as prolyl tripeptidyl‐peptidase A (PTP‐A), even though plasma proteins serving as potential nutrients for *P. gingivalis* are frequently N‐terminally modified. Second, polypeptides with Pro at the third position from the N‐terminus are resistant to DPPs.

The first issue is surmounted by acylpeptidyl‐oligopeptidase (AOP), which preferentially degrades polypeptides with N‐terminal modification into di‐ and tri‐peptides (Nemoto et al., 2016). The second one is also surmounted by PTP‐A, which liberates NH_2_‐Xaa1‐Xaa2‐Pro3 from polypeptides (Banbula et al., 1999; Ito et al., 2006). In other words, AOP and PTP‐A have possibly evolved to complete dipeptide production by DPPs in *P. gingivalis*.

### 3.1 AOP

AOP, which belongs to the S09 family, preferentially degrades N‐terminally acylated polypeptides to liberate acylated‐di‐ and tri‐peptides (Nemoto et al., 2016). The most potent synthetic substrate for *P. gingivalis* AOP is benzyloxycarbonyl‐Lys‐Met‐MCA. The structure of acylated compounds is not of concern, whereas concealment of the N‐terminal α‐amino group is essential for efficient degradation. Although acylaminoacyl‐peptidase (AAP), which specifically removes N‐terminal acylated amino acids from a polypeptide, has been previously reported in the human and archaea *Aeropyrum pernix* (Kiss et al., 2007), the amino acid sequence identity (15.8%) between *P. gingivalis* AOP and *Aeropyrum pernix* AAP is limited, while AOP scarcely removes acylated N‐terminal amino acids but dipeptides. Modeling of *P. gingivalis* AOP revealed the hydrophobic S1 site of AOP in accord with its hydrophobic P1 preference as well as an N‐anchor region determining the substrate specificity with a more open active site than those of DPPs accepting a broad range of N‐terminal different steric groups (Nemoto et al., 2016).

Comparisons of three AOPs from *P. gingivalis*, *Bacteroides dorei*, and *Lysinibacillu sphaericus* revealed that the activity of *P. gingivalis* AOP was most significantly enhanced by acylation of substrates, further supporting the removal of acylated N‐terminus as the primary role of that AOP (Nemoto et al., 2019).

### 3.2 PTP‐A

PTP‐A was first discovered in *P. gingivalis* and belongs to S9.017, and has been shown to hydrolyze the Pro3‐Xaa4 bond (Banbula et al., 1999). Reflecting the similarity of substrate specificities, the amino acid sequence of *P. gingivalis* PTP‐A is 23.5% identical to that of *P. gingivalis* DPP4. An unblocked N‐terminus is required for its activity and no cleavage occurs with substrates with Pro at the P1’ position. Synthetic substrates, Ala‐Ala‐Pro‐ and Gly‐Ala‐Pro‐*p*‐nitroanilide, are used for the assay of PTP‐A, and it has been proposed that PTP‐A is involved in the degradation of type I collagen in connection with periodontitis inflammation (Ito et al., 2006). However, because the Gly‐Xaa‐Pro repeat is located at the center of the collagen sequences, it is ambiguous whether this exopeptidase truly degrades collagens in host tissues. Hence, the primary role of PTP‐A is likely to provide oligopeptides without Pro at the third position from the N‐terminus for DPPs. Genome analysis (Naito et al., 2008) as well as biochemical studies up to date failed to find tripeptidyl‐peptidases except for PTP‐A, which strongly suggests the absence of other tripeptidyl‐peptidases in *P. gingivalis*.

### 3.3 FOUR DPPs, AOP, AND PTP‐A LOCATED IN PERIPLASM

The enzymatic activities of DPPs, AOP, and PTP‐A are scarcely detected in bacterial culture supernatant, indicating that they are cell‐associated. In addition, we previously showed the periplasmic localization of DPP5 by subcellular fractionation (Ohara‐Nemoto et al., 2014). Although the subcellular localization of other DPPs, AOP, and PTP‐A has not been biochemically verified, they possess typical signal sequences for export from the inner membrane but do not possess a conserved C‐terminal domain (CTD) of 70–80 amino acids, a prerequisite for secretion *via* the Type‐9 secretion system (T9SS) (Sato et al., 2010; Seers et al., 2006; Slakeski et al., 2011). In accord with signal sequence prediction of Met1‐Ala21 in *P. endodontalis* DPP11, the N‐terminal Asp22 was identified in an endogenous form (Ohara‐Nemoto et al., 2011). Accordingly, DPPs are transported through the inner membrane and localized in the periplasmic space. *P. gingivalis* AOP also possesses a potent signal sequence. Although the probability is less significant, Met1‐Ala38 in *P. gingivalis* PTP‐A may correspond to the signal peptide. Proteome analysis of *P. gingivalis* W50 also suggested the localization in the lumen of the vesicle (periplasm) of DPP4 (PG_0503), DPP5 (PG_1004), DPP7 (PG_0491), and DPP11 (PG_1283) (Veith et al., 2014). Moreover DPP4 seems to be is enriched in outer membrane vescicle under heme excess conditions (Veith et al., 2018). In contrast, DPP3 and DPP6 seem to be located in the cytoplasm, and thus, not involved in the degradation of extracellular proteins in the periplasm. Their roles should be elucidated in future studies.

**FIGURE 1:**
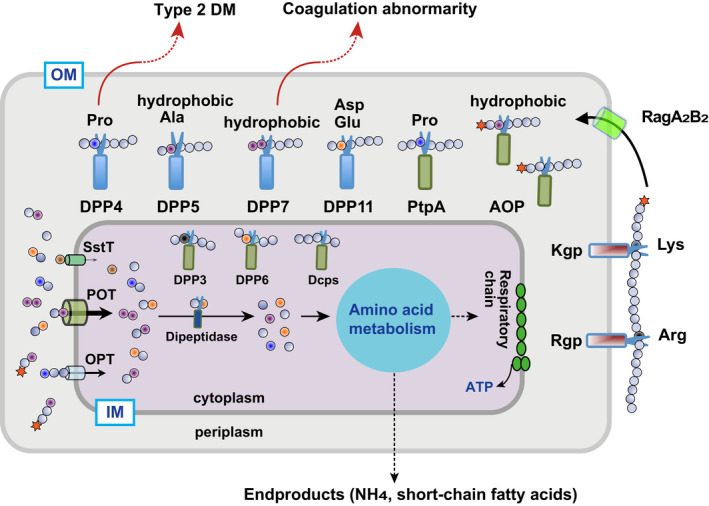
Schematic illustration on dipeptide production and incorporation in *Porphyromonas gingivalis*. OM and IM, outer and inner membranes, respectively [Colour figure can be viewed at wileyonlinelibrary.com]

Taken together, the degradation pathway of extracellular proteins in *P. gingivalis*, which is initiated by gingipains and finally reach dipeptides mediated by DPPs with the help of AOP and PTP‐A, is schematically illustrated in Figure 1. We currently suppose that oligopeptidyl substrates and dipeptidyl MCA are incorporated across the outer membrane via RagAB.

## 4 DIPEPTIDE INCORPORATION BY PROTON‐DEPENDENT OLIGOPEPTIDE TRANSPORTER POT

Genome information for *P. gingivalis* indicates the existence of a single copy of each of three amino acid/oligopeptide transporters, *i.e,* serine/threonine transporter (SstT), proton‐dependent oligopeptide transporter (POT), and oligopeptide transporter (OPT) (Naito et al., 2008; Nelson et al., 2003). Among them, only *P. gingivalis* SstT, which incorporates amino acids with most efficiently Ser and Thr, has been reported (Dashper et al., 2001). The analysis of transporter‐gene disrupted strains of *P. gingivalis* indicates that a Δ*pot* mutant of *P. gingivalis* had a significant defect in incorporation of dipeptides, while a Δ*opt* strain had a moderate decrease in several dipeptides (Ohara‐Nemoto et al., 2019). As expected, a Δ*sstT* strain did not exhibit a decrease in dipeptide incorporation. Moreover, the growth of the Δ*pot* strain was the most significantly retarded among the three single transporter‐deficient mutants, and the double mutant Δ*pot‐*Δ*opt* scarcely grew (Ohara‐Nemoto et al., 2019). These results indicated that nutritional dipeptides are primarily incorporated by POT in *P. gingivalis*.

It seems unusual that there is only a single copy of each of the three transporter genes in *P. gingivalis*, because there are multiple transporter paralogues in other bacteria. For example, *E. coli* possess four POT members (*ydgH*, *ydgR*, *yhiP*, *yidL*) (Ernst et al., 2009; Weitz et al., 2007), and *Helicobacter pylori*, limited to utilization of carbohydrates as carbon sources, has five POT (*dppA*, *B*, *C*, *D*, *F*) and four OPT (*oppA*, *B*, *C*, *D*) paralogs (Salama et al., 2000; Weinberg & Maier, 2007). The presence of multiple sets of transporter genes suggests that these bacteria readily respond to changes in nutritional conditions, while *P. gingivalis* resides in the consistently poor nutritional environment of subgingival dental plaque.

## 5 DIPEPTIDASES DEGRADE DIPEPTIDES INTO SINGLE AMINO ACIDS

Dipeptides incorporated into bacterial cells are cleaved by a dipeptidase to two amino acids, which enter the metabolic pathway of the cell (Figure 1). To date, little is known regarding dipeptidases in *P. gingivalis* and other periodontopathic bacteria. We recently identified Arg‐preferential dipeptidase A [locus tag, PIOMA14_1_1238, MEROPS code, BAU17746] in *Pre. intermedia*, which belongs to C69.001 (Sarwar et al., 2020). *Pre. intermedia* dipeptidase A most preferentially degrades Arg‐Leu and Arg‐Phe, and does other dipeptides, such as Leu‐Leu and Glu‐Glu, moderately. It never degrades tripeptides and does not show any amino peptidase ativity (Sarwar et al., 2020). Dipeptidase A/PepDA (MER0233043) from *Lactobacillus helveticus*, the representative enzyme of the C69.001 members, was reported to possess a hydrophobic P1 preference (Dudley et al., 1996; Vesanto et al., 1996). However, a later reexamination revealed that it also possesses an Arg preference. *Pre. intermedia* encodes one additional C69‐family member (BAU18827), though no hydrolyzing activity has been observed (Sarwar et al., 2020). *P. gingivalis* carries a single C69 family member, PGN_1103, of which the deduced sequence is 13.1% and 47.3% identical to that of BAU17746 and BAU18827 of *Pre. intermedia*, respectively. Therefore, PGN_1103 is likely to be an ortholog of BAU18827 and again results of our preliminary search of PGN_1103 revealed no dipeptidase activity present.

## 6 OTHER POTENTIAL EXOPEPTIDASES INVOLVED IN DIPEPTIDE PRODUCTION

Genome information demonstrates the presence of three additional uncharacterized S9‐family members; PGN_1542 (annotated S9 unassigned), PGN_1694 (S9 unassigned), and PGN_1878 (Ala‐DPP of S9C), of which the deduced sequences are predicted to possess signal sequences, Met1‐Ala21, Met1‐Ala20, and Met1‐Ser20, respectively, indicating their periplasm localization. They have been expressed as recombinant proteins, though their peptidase activities have yet to be identified (Nemoto et al., 2016; Ohara‐Nemoto et al., 2011). Peptidyl‐dipeptidase (Dcp) is an M3‐family exopeptidase that liberates C‐terminal dipeptides from longer polypeptides (Paschoalin et al., 2013) and when present, efficiency for producing dipeptides should be facilitated. *P. gingivalis* truly possesses two potential Dcp genes, PGN_1776 (MER0034589) and PGN_0788 (MER003588), though they have no signal sequences. Accordingly, these potential Dcps may be cytosolic proteins and are unlikely to be involved in dipeptide production in the periplasm.

### 6.1 *P. GINGIVALIS* DPPs RELATING TO SYSTEMIC DISEASES

Currently, the two‐way relationship between periodontal disease and type 2 diabetes mellitus is accounted for by chronic inflammation, in which an elevated level of tumor necrosis factor‐α derived from the liver by lipopolysaccharide of periodontopathic Gram‐negative bacteria, results in insulin resistance (Soorya et al., 2014; Takano et al., 2010; Teeuw et al., 2010). We recently proposed that an additional and direct link exists between the two diseases, in which periodontopathic bacterial DPP4 degrades incretins (Ohara‐Nemoto et al., 2017). Since incretins are multifunctional (Seino & Yabe, 2013) and other bioactive peptides including gastrointestinal hormones, neuropeptide, and chemokines could be potential targets, periodontopathic bacterial DPPs may modulate human homeostasis *via* inactivation of these molecules.

### 6.2 DPP4 as a modulator for type 2 diabetes mellitus

Human DPP4 cleaves the peptide bond between the second Ala and third Glu of GLP‐1 and GIP, causing rapid inactivation (Mentlein et al., 1993). Hence, DPP4 inhibitors are widely used to treat type 2 diabetes mellitus patients. Because of the common enzymatic features, the involvement of periodontopathic bacterial DPP4 in modulation of blood glucose level is reasonably inferred.

*Porphyromonas gingivalis* cells were found to efficiently degrade GLP‐1 and GIP, while a Δ*dpp4* mutant did not. In a glucose tolerance test, intravenous injection into mice of recombinant DPP4 from *P. gingivalis*, *T. forsythia*, and *Pre. intermedia* reduced the plasma GLP‐1 active form and insulin levels, which were accompanied by a substantial elevation in postprandial hyperglycemia together with retardation of the decrease in blood glucose levels (Ohara‐Nemoto et al., 2017). Since oral bacteremia occurs as a result of mastication, toothbrushing, and dental procedures (Tomás et al., 2012), the higher incidence of periodontopathic bacteremia in individuals suffering from severe periodontal disease seems to exacerbate type 2 diabetes mellitus through degradation of incretins by bacterial DPP4 in the bloodstream. Oral bacteria that form subgingival biofilm possess DPP4 orthologues, thus may also participate in this phenomenon. Because human DPP4 inhibitors are less efficient for bacterial DPP4 (Rea et al., 2017), development of periodontopathic DPP4‐specific inhibitors and the usage for type 2 diabetes mellitus patients may synergistically reduce blood glucose concentration.

### 6.3 DPP7 as a virulence factor of hemolysis

Infection by *C. canimorsus* caused by a severe dog or cat bite occasionally induces bleeding and coagulation abnormalities. Hack et al. (2017) reported that*. C. canimorsus* DPP7, which degrades and inactivates coagulation factor X at the N‐termini of its light and heavy chains, is responsible for these conditions. Their study strongly suggests that *P. gingivalis* and *Capnocytophaga gingivalis* DPP7 may retard the prothrombin time and activated partial thromboplastin time. This capability of DPP7 should be profitable for periodontopathic bacteria, because retardation of coagulation may provide hemoglobin and plasma proteins as nutrition sources for periodontopathic asaccharolytic bacteria. This issue should be further investigated in the future.

## 7 CONCLUDING REMARKS

*Porphyromonas gingivalis*, an asaccharolytic Gram‐negative rod, utilizes extracellular proteins as its sole carbon and energy sources. Since nutritional polypeptides are mainly incorporated as dipeptides in bacterial cells, they should be initially degraded into oligopeptides by endopeptidases, and Lys‐ and Arg‐gingipains located at the outer membrane, and then degraded into dipeptides by DPP4, DPP5, DPP7, and DPP11. Two exopeptidases, AOP and PTP‐A, function to transform oligopeptides into forms cleavable by DPPs. Dipeptides are incorporated in the cells mainly by the POT transporter. Bacterial DPP4 and DPP7 are closely related to human systemic diseases. Since multifunctional incretin peptides and other bioactive peptides, including gastro‐intestinal hormones, neuropeptides, and chemokines, are potent targets, periodontopathic bacterial DPPs are psossively involved in modulation of human homeostasis *via* degradation of these molecules. In conclusion, proteins involved in dipeptide production and incorporation have emerged as potent targets in therapy for periodontal and related systemic diseases.

## CONFLICT OF INTEREST

None to declare.

### Peer Review

The peer review history for this article is available at https://publons.com/publon/10.1111/omi.12317.
